# Gender-specific differences in PPARγ regulation of follicular helper T cell responses with estrogen

**DOI:** 10.1038/srep28495

**Published:** 2016-06-23

**Authors:** Hong-Jai Park, Hyeon-Soo Park, Jae-Ung Lee, Alfred L. M. Bothwell, Je-Min Choi

**Affiliations:** 1 Department of Life Science, College of Natural Sciences, Hanyang University, Seoul, 133-791, Republic of Korea; 2 Research Institute for Natural Sciences, Seoul, 133-791, Republic of Korea; 3 Center for Neuroscience Imaging Research (CNIR), Institute for Basic Science (IBS), Suwon 440-746, Republic of Korea.; 4 Department of Immunobiology, Yale University School of Medicine, New Haven, CT 06520, USA.

## Abstract

Peroxisome proliferator-activated receptor gamma (PPARγ), a master regulator of adipocyte differentiation, has recently been connected with effector T cells, though its role is still not clear. Here, we investigated the roles of PPARγ in follicular helper T (T_FH_) cell responses regarding gender specificity. NP-OVA immunization in female but not male CD4-PPARγ^KO^ mice induced higher proportions of T_FH_ cells and germinal center (GC) B cells following immunization than were seen in wild type mice. Treatment with the PPARγ agonist pioglitazone significantly reduced T_FH_ cell responses in female mice while pioglitazone and estradiol (E2) co-treatment ameliorated T_FH_ cells and GC responses in male mice. E2 treatment significantly enhanced PPARγ expression in male T cells, while T cell activation in the estrus but not in the diestrus stage of the menstrual cycle of females was inhibited by pioglitazone, suggesting that an estrogen-sufficient environment is important for PPARγ-mediated T cell regulation. These results demonstrate gender-based differences in sensitivities of PPARγ in T_FH_ responses. These findings suggest that appropriate function of PPARγ is required in the regulation of female GC responses and that therapeutic strategies for autoimmune diseases using PPARγ agonists need to be tailored accordingly.

PPARγ is a transcription factor and a master regulator of adipocyte differentiation^1^,^2^,^3^,^4^,^5^. It is activated by ligands such as 15-deoxy-Δ^12,14^-prostagladin J_2_ (15d-PGJ2)^6^,^7^ and 13-hydroxyoctadecadienoic acid (13-HODE)^8^, which are derived from eicosanoids including prostaglandin D_2_ or fatty acid metabolites^9^. Thiazolidinediones (TZDs) such as pioglitazone, rosiglitazone, ciglitazone, and troglitazone are synthetic ligands for PPARγ^10^, and have been approved for use in the treatment of type 2 diabetes mellitus^11^. These ligands effectively inhibit NF-kB function to regulate inflammation and inflammatory diseases^12^.

PPARγ has been highlighted in T cell responses and autoimmune diseases and PPARγ ligand treatment has been shown to inhibit effector T cell functions *in vitro* and *in vivo*. Ciglitazone and 15d-PGJ2 inhibited T cell proliferation and IL-2 production^13^. We previously reported that pioglitazone inhibited human memory T cell responses in a model of arterial grafts^14^. Pioglitazone was also reported to selectively regulate Th17 cell responses to ameliorate experimental autoimmune encephalomyelitis (EAE)^15^. More recently, PPARγ-deficient Treg cells showed an impaired ability to migrate to visceral adipose tissue on a high-fat diet^16^ and failed to regulate effector T cell functions and development of colitis and GVHD^17^,^18^. In contrast, another study showed that PPARγ contributed to the development of colitis in a lymphopenic environment^19^. Thus, there are still some controversies regarding the role of PPARγ in T cells.

Previously, we reported that female CD4-PPARγ^KO^ mice have spontaneous autoimmune phenotypes with increased T_FH_ cells and GC reactions^20^. Here, we observed that male CD4-PPARγ^KO^ mice do not develop autoimmune phenotypes and hypothesized that there are gender-based differences in PPARγ regulation of T_FH_ cell responses. In this report, we demonstrate that an estrogen-sufficient environment promotes PPARγ activity to regulate T_FH_ responses and we also suggest that PPARγ is more necessary in females to regulate effector T cell responses than it is in males.

## Results

### Female CD4-PPARγ^KO^ but not male mice induce a higher proportion of T_FH_ cells and GC B cells

**Figure 1:**
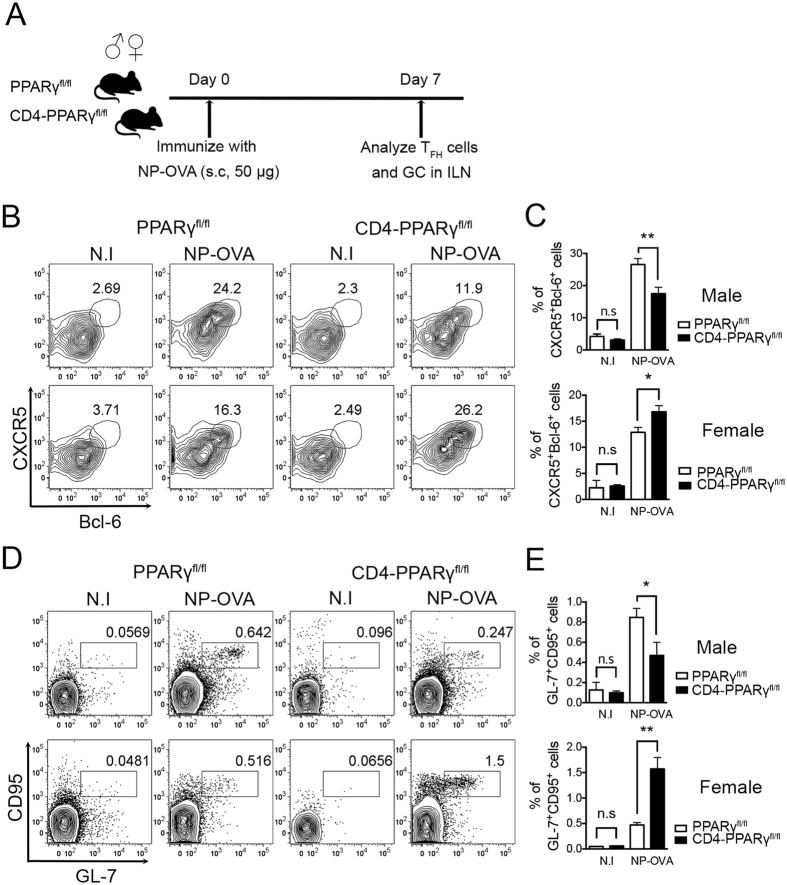
Female CD4-PPARγ^KO^ but not male mice have higher proportions of T_FH_ cells and GC B cells. (**A**) T_FH_ cells were induced by immunization of six- to eight-week-old male and female littermate control and CD4-PPARγ^KO^ mice with NP-OVA. Seven days after immunization, the proportion of T_FH_ cells was analyzed by flow cytometry. (**B**,**C**) NP-OVA-immunized male and female littermate control and CD4-PPARγ^KO^ mice were analyzed by staining with anti-Bcl-6 and anti-CXCR5 antibodies. Bcl-6 and CXCR5 double-positive cells gated on CD4^+^CD44^high^ were identified as T_FH_ cells and the % of Bcl-6^+^CXCR5^+^ T_FH_ cells was represented as a bar graph. (**D**,**E**) Germinal center (GC) B cells in male and female littermate control and CD4-PPARγ^KO^ mice were examined by staining with anti-GL-7 and anti-CD95 antibodies seven days after NP-OVA immunization and the % of GL-7^+^CD95^+^ GC B cells gated on B220-positive cells was indicated as a bar graph. The data represent means ± SEM (n = 4/group, three independent experiments). *P < 0.05, **P < 0.01 by a two-tailed, unpaired Student’s *t*-test.

Previously, we reported that female CD4-PPARγ^KO^ mice spontaneously develop autoimmune phenotypes with an increase in T_FH_ cells with GC responses^20^. However, interestingly, 6-month to 1-year-old male CD4-PPARγ^KO^ mice did not show spontaneous autoimmune phenotypes including the proportion of activated T cells (Supplementary Fig. S1a,b), autoantibodies in the serum (Supplementary Fig. S1c,d), and glomerulus inflammation (Supplementary Fig. S1e,f), which are typical indicators of systemic autoimmune diseases. In addition, there was no difference in the proportion of T_FH_ cells in the spleen (Supplementary Fig. S1g,h). These results prompted us to consider the possibility of gender-based differences in phenotypic sensitivity of PPARγ deletion in T cells. To determine gender-based differences in T_FH_ responses to antigen immunization in CD4-PPARγ^KO^ mice, we first immunized six- to eight-week-old mice with NP-OVA (4-Hydroxy-3-nitrophenyl-ovalbumin) and analyzed T_FH_ cells and GC responses in the draining lymph node seven days after the immunization (Fig. 1A). CXCR5 and Bcl-6 double-positive cells gated on CD4^+^CD44^high^ were identified as T_FH_ cells^21^,^22^,^23^. The proportion and the absolute cell number of T_FH_ cells was significantly increased in female CD4-PPARγ^KO^ mice compared with the wild-type littermate control group, while there was a significant decrease in T_FH_ cells in the draining lymph node of male CD4-PPARγ^KO^ mice (Fig. 1B,C, Supplementary Fig. S2a). In addition, female CD4-PPARγ^KO^ mice displayed a significantly increased population and the cell number of of GL-7^+^CD95^+^ GC B cells, while male CD4-PPARγ^KO^ mice showed a population of GC B cells smaller than that of the control group (Fig. 1D,E, Supplementary Fig. S2b). These data demonstrate that there are gender-specific effects of PPARγ deletion in T cells, and PPARγ is required to prevent T_FH_ responses in female but not in male mice.

### PPARγ agonist pioglitazone treatment reduces T_FH_ responses in female but not in male mice

**Figure 2:**
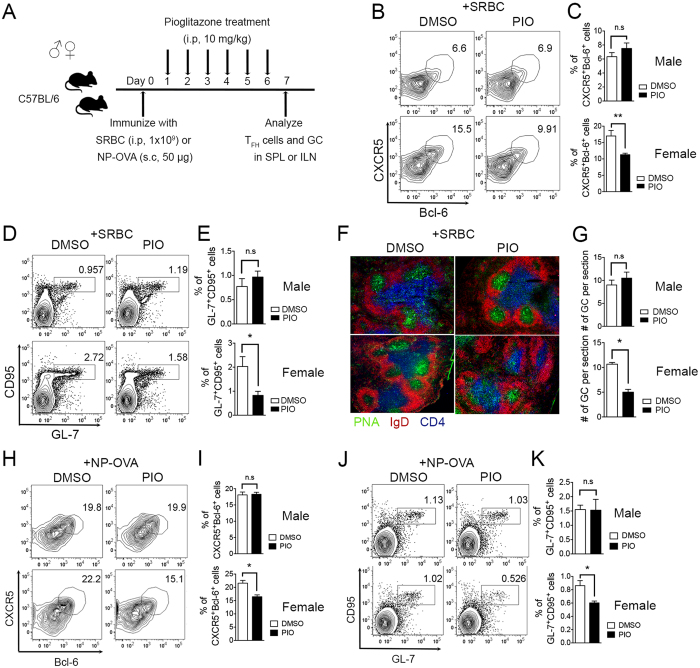
PPARγ ligand pioglitazone treatment reduces T_FH_ responses in female but not in male mice. (**A**) Six- to eight-week-old male and female mice were immunized with SRBC or NP-OVA and were treated daily with pioglitazone (10 mg/kg) intra-peritoneally from day 1 to day 6. (**B**,**C**) The mice were sacrificed following SRBC immunization and the spleens were isolated and stained with anti-mouse CXCR5 and Bcl-6 antibodies to analyze the proportion of T_FH_ cells and the % of CXCR5^+^Bcl-6^+^-positive T_FH_ cells was represented as a bar graph. (**D**,**E**) GL-7 and CD95 double-positive cells gated on B220-positive cells from the spleens from male and female mice were analyzed and the % of GL-7^+^CD95^+^ GC B cells was demonstrated as a bar graph (n = 3/group, three independent experiments). (**F**,**G**) Immunofluorescence analysis was performed to determine the number of GCs formed in spleens of SRBC-immunized male and female mice by staining with anti-PNA, -IgD, and -CD4 antibodies and the numbers of GCs were counted per spleen section. The average number of GCs per spleen section was determined (n = 9). (**H,I**) The inguinal lymph nodes were isolated from NP-OVA-immunized male and female mice. CXCR5^+^Bcl-6^+^-positive T_FH_ cells were analyzed and displayed as a bar graph. (**J,K**) GL-7 and CD95 double-positive cells gated on B220-positive cells in the inguinal lymph nodes from male and female mice were analyzed and the % of GL-7^+^CD95^+^ GC B cells was represented as a bar graph. The data shown represent means ± SEM (n = 3 per group, two independent experiments). *P < 0.05, **P < 0.01 by a two-tailed, unpaired Student’s *t*-test.

To determine whether PPARγ agonist pioglitazone treatment also shows gender-based differences in its effects on the induction of T_FH_ cells and GC responses, six- to eight-week-old C57BL/6 mice were immunized with SRBC or NP-OVA, 10 mg/kg of pioglitazone was administered intra-peritoneally once a day from day 1 to day 6, and T_FH_ cells and GC responses were analyzed in the spleen or draining lymph node at day 7 (Fig. 2A). We observed that pioglitazone treatment significantly inhibited the proportion of T_FH_ cells in the spleen compared to the DMSO-treated control group only in female mice, while no effect was observed in the males (Fig. 2B,C). The population of GC B cells was also significantly diminished by pioglitazone treatment in females, whereas it was not affected in male mice (Fig. 2D,E). Frozen spleen tissue was sectioned and stained to determine the T cell zone (anti-CD4-APC, blue), B cell zone (anti-IgD-PE, red), and GCs (PNA, green). The number of GCs was significantly reduced in female mice, but not in males following pioglitazone treatment (Fig. 2F,G). These observations are consistent with those in a NP-OVA immunization model showing that pioglitazone treatment inhibited a proportion of T_FH_ cells and GC cells of the draining lymph node only in female mice (Fig. 2H–K). These results collectively demonstrate that the stimulation of PPARγ via its ligand also has gender-based differences in effects in that it significantly inhibits T_FH_ responses only in female mice but is not effective in males.

### Pioglitazone and estradiol co-treatment in males reduces T_FH_ responses

**Figure 3:**
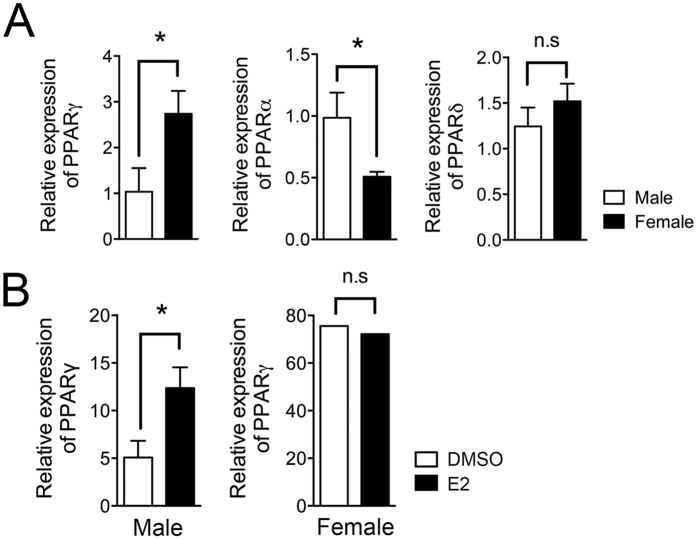
Estradiol treatment enhances the PPARγ expression. (**A**,**B**) Total RNA was isolated from male and female naïve T cells (CD4^+^CD62L^high^) to determine the PPAR expression levels. Basal expression of PPARs in male and female naive T cells and PPARγ expression in 5 nM E2- or DMSO-treated male and female naïve T cells following TcR stimulation for 3 days were assessed using real-time PCR and were normalized to β-actin. *P < 0.05 by two-tailed, unpaired Student’s *t*-test. Values shown are means ± SEM (n = 3).

**Figure 4:**
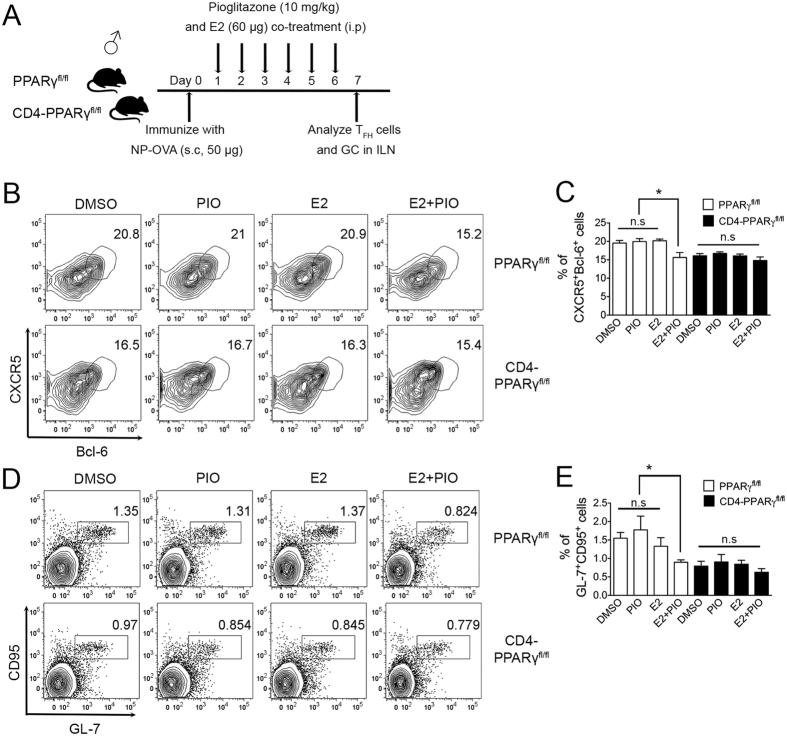
Pioglitazone and estradiol co-treatment in males reduces T_FH_ responses. (**A**) Six- to eight-week-old male CD4-PPARγ^KO^ mice and the littermate control mice were immunized with NP-OVA and were treated daily with DMSO, pioglitazone (10 mg/kg), E2 (60 μg), or pioglitazone (10 mg/kg) + E2 (60 μg) intra-peritoneally once a day from day 1 to day 6. (**B**,**C**) The mice were sacrificed on day 7 and inguinal lymph nodes were isolated and the cells were stained with anti-mouse CXCR5 and Bcl-6 antibodies to analyze the proportion of T_FH_ cells and the % of CXCR5^+^Bcl-6^+^-positive T_FH_ cells was indicated as a bar graph. (**D**,**E**) GL-7 and CD95 double-positive cells gated on B220-positive cells from the lymph nodes were analyzed and the % of GL-7^+^CD95^+^ GC B cells were represented as a bar graph. The data shown represent means ± SEM (n = 5/group, two independent experiments). One-way ANOVA was used for statistical analysis. *P < 0.05.

From the gender-specific results regarding T_FH_ responses with PPARγ deletion in T cells or stimulation of PPARγ by its ligand following immunization, we hypothesized that estrogen in the female might be important for this action of PPARγ. We first measured the basal PPARγ mRNA expression levels in male and female MACS-purified naïve T cells (CD4^+^CD62L^high^) and also determined whether E2 treatment enhances PPARγ levels in male T cells. From the results, we found that PPARγ mRNA was expressed at significantly higher levels in female T cells than in males while male T cells had higher levels of PPARα and comparable expression of PPARδ (Fig. 3A). Interestingly, 5 nM E2 treatment of male T cells augmented PPARγ mRNA expression compared with DMSO-treated T cells, while no altered expression was found in E2-treated female T cells (Fig. 3B) suggesting that there is some positive feedback action of estrogen on PPARγ expression. To investigate the synergistic role of E2 and pioglitazone, six- to eight-week-old male C57BL/6 mice and CD4-PPARγ^KO^ mice were immunized with NP-OVA and treated with either 60 μg of E2 and/or 10 mg/kg of pioglitazone once a day from day 1 to day 6, then T_FH_ cells and GC responses in the draining lymph node were analyzed seven days after immunization (Fig. 4A). *In vivo* administration of E2 for six days results in significantly increased PPARγ mRNA expression in the spleen of male mice which is comparable level in estrus cycle of female mice (Supplementary Fig. S3). Only co-treatment with pioglitazone and E2, and not either treatment by itself, significantly inhibited the proportion of T_FH_ cells in the lymph node compared to the other groups in male mice (Fig. 4B,C). The proportion of GC B cells was also significantly reduced by pioglitazone and E2 co-treatment (Fig. 4D,E). The lack of any effect of this co-treatment in CD4-PPARγ^KO^ mice suggests that the co-treatment effect is dependent on PPARγ action. These results collectively suggest that E2 enhances PPARγ sensitivity in male mice for the regulation of T_FH_ responses.

### Pioglitazone inhibits T cell activation in the estrus but not in the diestrus stage of the menstrual cycle in females

**Figure 5:**
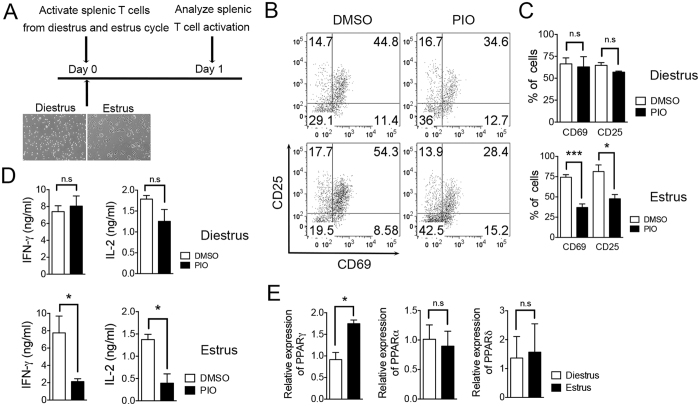
Pioglitazone inhibits T cell activation in the estrus but not in the diestrus stage of the menstrual cycle in females. (**A**) Menstrual cycle was monitored in six- to eight-week-old female C57BL/6 mice and splenocytes were isolated from the mice at estrus and diestrus stages. The cells were activated with anti-CD3 and anti-CD28 antibodies for 24 h in the presence of DMSO or pioglitazone. (**B**,**C**) CD69 and CD25 expression gated on CD4-positive cells were analyzed with flow cytometry and the % of CD69- and CD25-positive cells were represented. (**D**) IFN-γ and IL-2 cytokine production levels following TcR stimulation were analyzed by ELISA using cultured supernatant. (**E**) Total RNA was isolated from female splenocytes from diestrus and estrus stages and the expression level of PPARs was analyzed by real-time PCR and normalized to β-actin. The data shown represent means ± SEM (n = 5/group). *P < 0.05, ***P < 0.001 by a two-tailed, unpaired Student’s *t*-test.

Due to the dynamic estrogen cycle in females, we hypothesized that PPARγ sensitivity in T cells might also differ during the menstrual cycle of female mice. We isolated splenocytes during the estrus and diestrus stages and then stimulated the cells with anti-CD3 and CD28 antibodies followed by pioglitazone treatment to determine if the differential PPARγ sensitivity depends on estrogen level (Fig. 5A). The levels of activation markers, CD25 and CD69, in CD4^+^ T cells were significantly reduced by pioglitazone treatment in the cells only at estrus cycle but not at diestrus cycle (Fig. 5B,C). In addition, production of IFN-γ and IL-2 by activated splenocytes was also significantly decreased by pioglitazone only at the estrus stage of the cycle (Fig. 5D). These results seem to correlate with PPARγ expression level since the cells from the estrus stage have higher expression levels of PPARγ than cells from the diestrus stage (Fig. 5E). As consistent with previous results, pioglitazone treatment could not inhibit T cell activation in male splenocytes (Supplementary Fig. S4a–c). Taken together, these results suggest that estrogen level has a positive correlation with PPARγ sensitivity to its ligand in females, thereby regulating T cell responses.

## Discussion

PPARγ is a master regulator in adipocyte differentiation, which has important roles in lipid metabolism. PPARγ has been recently studied in T cells where it was shown to regulate Th17 cells to prevent autoimmunity and was also found to be necessary for regulatory T cell functions. Here, we demonstrate gender-specific actions of PPARγ regarding effector T cell functions, such as T_FH_ responses, which are supported by estrogen.

Previously, it was reported that treatment with the PPARγ agonist pioglitazone augmented the incidence of hypoglycemia in diabetic women^24^ and that rosiglitazone showed a greater reduction of fasting plasma glucose (FPG) levels in women than in men^25^, suggesting that women have a greater sensitivity to treatment with PPARγ ligands. This gender-based difference in sensitivity would be correlated to the sex-dimorphic expression of PPARγ. Previously, a sex-specific action of PPARα was also reported when it was observed that male mice lacking PPARα were more susceptible to EAE than were female mice and that PPARα expression level was higher in male T cells than in female T cells^26^. In addition, PPARα siRNA treatment affected only human male T cells by enhancing IFN-γ production, while there was no difference in IFN-γ expression in female T cells. PPARα ligand fenofibrate diminished IFN-γ production in males, but not in females^27^. Fenofibrate was also reported to reduce body weight and white adipose tissue (WAT) mass in high-fat-diet-fed male mice, but not in females^28^, suggesting that PPARα and PPARγ have gender-based specific sensitivities in their biological roles.

In general, women are more susceptible to autoimmune diseases compared to men^29^,^30^,^31^. Several factors including hormones and the X-chromosome have been suggested to affect the higher prevalence rates of autoimmune diseases in females. We showed here that PPARγ expression is higher in T cells from female mice than in cells from males and that pioglitazone effects have a positive correlation with estrogen levels. Here we suggest that PPARγ controls T_FH_ responses more sensitively in females with sufficient estrogen levels. This finding suggests that an abnormality in PPARγ activity in T cells might cause a more critical problem for maintaining homeostasis in females than in males. T_FH_ cells are found in B cell follicles and interact with cognate B cells to promote isotype class switching, affinity maturation, and plasma cell differentiation to produce antibodies^32^,^33^. Therefore, T_FH_ cells and GC reactions are important drivers of autoimmune disease by supporting autoantibody production^34^. Regulation of T_FH_ cells is now considered to be an important target for treatment of autoimmune diseases^35^. We demonstrate that PPARγ plays a significant role in the regulation of T_FH_ responses, especially in females, which would be important to prevent autoimmunity.

Decreased numbers of T_FH_ cells in male CD4-PPARγ^KO^ mice following NP-OVA immunization were observed in our experiments, raising the possibility that the discrepancy in PPARγ function acts as a negative regulator of effector T cell functions, although pioglitazone treatment has no effect in males. One recent paper reported that PPARγ is required for the development of autoimmunity in lymphopenic conditions due to increased apoptosis with reduced IL-7Rα expression of CD4-PPARγ^KO^ T cells^19^, while there is a controversial result from previous studies showing that PPARγ is a negative regulator of T cell activation. It was reported that the transfer of PPARγ-deficient effector T cells into RAG-knockout mice showed a robust induction of colitis^17^. In addition, PPARγ deficiency in T cells showed increased Th17 and EAE disease pathogenesis and pioglitazone treatment selectively inhibited Th17 factors, suggesting a role for this drug in suppressing Th17 differentiation^15^. More recently, Treg-specific PPARγ deficiency showed an abnormality on Treg migration into adipose tissue in a high-fat diet animal model^16^. We hypothesize that this discrepancy could result from the different genders of the mice used. We determined that male PPARγ-deficient T cells have reduced levels of Bcl-2 and IL-7Rα expression, which are critical for T cell survival, while there is no difference in female CD4-PPARγ^KO^ mice compared to their controls (Supplementary Fig. S5a,b). We also found that male PPARγ-deficient T cells are more apoptotic than wild-type controls in serum starvation conditions (Supplementary Fig. S5c,d). Therefore, PPARγ in males contributes to the survival of T cells by maintaining Bcl-2 and IL-7Rα expression while an estrogen-sufficient environment might compensate to sustain anti-apoptotic molecular expression in females^36^. Recently, role of IL-7Rα signaling in the regulation of T_FH_ cells has been suggested that IL-7 suppressed the expression of Bcl-6 and other T_FH_ genes^37^. In addition, IL-7Rα-STAT5 axis is suggested as a negative regulator of T_FH_ responses like IL-2-STAT5^38^. However, another previous study regarding Foxo1 deletion in T cells with transgenic of IL-7Rα expression demonstrated that alteration of T_FH_ responses are dependent on Foxo1 but not IL-7Rα^39^. Thus, possible reason for reduced induction of T_FH_ cells without PPARγ in males need to be further investigated regarding decreased IL-7Rα and Bcl-2 levels.

Synthetic ligands for PPARγ are used as anti-diabetes drugs for the treatment of type II diabetes mellitus^40^,^41^,^42^,^43^,^44^. Those ligands are also effective in the regulation of autoimmune diseases including colitis and EAE. Troglitazone and rosiglitazone treatment remarkably reduced disease severity in a mouse colitis model by inhibiting activation of NF-κB^45^, while pioglitazone selectively inhibited Th17 cells to ameliorate the clinical features of EAE^15^. In addition to the synthetic ligands, various types of polyunsaturated fatty acid (PUFA) metabolites can serve as endogenous ligands for PPARγ to ameliorate inflammatory responses and autoimmune diseases. Previous studies have reported that PUFA metabolites, including eicosanoids and linoleic acid, modulated upregulation of PPARγ, resulting in reduced inflammation and IFN-γ production^46^,^47^. Animal models of inflammatory bowel diseases displayed significantly reduced colonic inflammation with PUFA feeding^48^ and reduced severity of EAE after PUFA supplementation^49^, suggesting that PPARγ has a potential role in the treatment of autoimmune diseases. Dietary intake of these metabolites could be an advantageous strategy, especially for females, to prevent autoimmune diseases by stimulating PPARγ to regulate sensitive T_FH_ responses.

Estrogen is the predominant sexual hormone in females and several studies have demonstrated the anti-inflammatory^50^ and protective role of E2 in an EAE model^51^,^52^. Due to the protective role of estrogen in autoimmune diseases, the incidence and severity of autoimmune diseases are worse in postmenopausal patients^53^, suggesting that the estrogen signal is essential to preventing autoimmunity. In our study, pioglitazone treatment inhibits activation of splenic T cells only when cells were collected during the estrus cycle and E2 treatment increased the PPARγ expression level in male T cells, suggesting that estrogen and PPARγ have a positive correlation in the regulation of T cell response. We did not observe any effect of E2 treatment alone on the regulation of T_FH_ cells or GC responses, suggesting that E2 enhances PPARγ level to increase the sensitivity to its ligand. Therefore, estrogen hormonal imbalance in females might result in abnormal control of PPARγ action to regulate T cells, which could contribute to sensitive auto-antibody production via T_FH_ responses. Molecular mechanisms of estrogen on PPARγ regulation and estrogen receptor deficiency in T cells will be further investigated regarding gender-specific regulatory mechanisms for T_FH_ responses.

In conclusion, our findings suggest that there is gender-specific sensitivity of PPARγ in the regulation of T_FH_ responses and that PPARγ-mediated regulation requires the estrogen signal in mice. Gender-based differences in therapeutic strategies using PPARγ agonists and combination treatments with estrogen should be considered for the treatment of autoimmune diseases.

## Materials and Methods

### Mice

B6.129-Pparg^tm2Rev^/J (PPARγ^fl/fl^) were purchased from the Jackson Laboratory (Bar Harbor, ME, USA). CD4-Cre^+/−^ transgenic mice were crossed with PPARγ^fl/fl^ mice to generate CD4-specific PPARγ-knockout mice (CD4-PPARγ^KO^). Mice were maintained at the Hanyang University mouse facilities under pathogen-free conditions with *ad libitum* feeding. All animal protocols in this study were approved by the Animal Experimentation Ethics Committee of Hanyang University and experiments were performed according to the guidelines of the Institutional Animal Care and Use Committees (IACUC) of Hanyang University.

### SRBC and NP-OVA immunization

Mice were immunized intra-peritoneally (i.p.) with sheep red blood cells (Innovative Research, Novi, MI, USA) diluted with DPBS at a 1:1 ratio and subcutaneously with 100 μg of NP-OVA (Bioresearch Technologies, Novato, CA, USA). Seven days after immunization, mice were sacrificed and spleens and inguinal lymph nodes were isolated and analyzed by flow cytometry and confocal microscopy. For PPARγ agonist treatment, pioglitazone was purchased from Sigma and dissolved in DMSO. To assess the regulatory effect of pioglitazone on T_FH_ cell differentiation *in vivo*, 10 mg/kg of pioglitazone was injected i.p. daily from day 1 to day 6 and the lymph nodes were isolated from the mice for further analysis.

### Flow cytometry

Splenocytes, mesenteric, and inguinal lymph node cells were isolated and then stained with anti-mouse CD4-APC, CD8-PerCP-Cy5.5, CD44-PE, CD62L-FITC, GL-7-FITC, CD95-PE, and B220-PerCP-Cy5.5 antibodies (eBioscience, San Diego, CA) for 15 min at 4 °C. For T_FH_ differentiation analysis, the cells were stained with anti-mouse CXCR5-biotin for 30 min at 4 °C followed by anti-mouse CD44-FITC, CD4-PerCP-Cy5.5, and streptavidin-APC staining. After fixation and permeabilization using the Foxp3 Staining Kit (eBioscience), anti-mouse Bcl-6-PE was stained for 1 h at room temperature. Cells were examined using the FACSCanto II system (BD Bioscience, San Jose, CA, USA) and data were analyzed using Flow Jo software (Treestar, Ashland, OR, USA). In all cases, doublets (FSC-area versus FSC-height gating) were excluded.

### RNA isolation and real-time PCR

RNA was isolated by a RNeasy mini kit (Qiagen, Valencia, CA, USA) according to the manufacturer’s protocol. RNA yield and purity were determined by NanoDrop. Total RNA (500 ng) was used for cDNA synthesis in a 20-μl reaction volume using qPCR RT Master Mix (Toyobo, Japan). Real-time PCR was performed using iQ SYBR Green Supermix (Bio-Rad, Hercules, CA, USA). Actin was used as a control housekeeping gene. The following primer sequences were used (forward/reverse): PPARγ, 5′-CTCCAAGAATACCAAAGTGCGA-3′ and 5′-GCCTGATGCTTTATCCCCACA-3′; Actin, 5′-TGTCCCTGTATGCCTCTGGT-3′ and 5′-CACGCACGATTTCCCTCTC-3′.

### Immunofluorescence

For GC formation analysis, the spleens from six- to eight-week-old sheep red blood cell (SRBC)-immunized mice were isolated 7 days after immunization and frozen in OCT compound. Tissues were sectioned to a 7-μm thickness, fixed in acetone at −20 °C, washed with PBS, and blocked with 0.1% BSA containing PBS for 30 min at room temperature. Tissues were stained with anti-PNA-FITC (Sigma-Aldrich, St. Louis, MO, USA), IgD-PE, and CD4-APC (eBioscience) antibodies diluted in blocking solution overnight at 4 °C. After three washes, tissues were incubated with ProLong Gold anti-fade reagent (Invitrogen, Life Technologies, Carlsbad, CA, USA) for 30 min at 4 °C and images were obtained using a Leica DM IRE2 confocal microscope.

### ELISA

Cytokine production in activated T cells and Th1, Th2, and Th17 cells was measured by ELISA using mouse IL-4, IL-13, and IL-17 Ready-SET-Go kits (eBioscience) and IFN-γ and IL-2 ELISA Deluxe sets (BioLegend, San Diego, CA, USA) according to the manufacturers’ instructions. Anti-dsDNA antibody in mouse serum was quantified by ELISA (Alpha Diagnostic International Inc, San Antonio, TX, USA).

### Statistical analysis

Data were analyzed statistically with the Student’s t-test and multiple comparisons were analyzed with one-way ANOVA using Prism5 (GraphPad, San Diego, CA, USA). P-values (P) less than 0.05 were considered statistically significant.

## Additional Information

**How to cite this article**: Park, H.-J. *et al*. Gender-specific differences in PPARγ regulation of follicular helper T cell responses with estrogen. *Sci. Rep.* **6**, 28495; doi: 10.1038/srep28495 (2016).

## Supplementary Material

Supplementary Information
