# Supplementary material for: Gender-specific differences in PPARγ regulation of follicular helper T cell responses with estrogen
Source: Sci Rep. 2016 Jun 23;6:28495. doi: 10.1038/srep28495 (PMC4917844; doi:10.1038/srep28495)
Supplement: Supplementary Information [file srep28495-s1.pdf]

Supplementary Information

Gender-specific differences in PPARγ regulation of follicular helper T cell responses with estrogen

Hong-Jai Park[1,2], Hyeon-Soo Park[1,2], Jae-Ung Lee[1,2], Alfred L. M. Bothwell[3], Je-Min Choi[1,2,*]

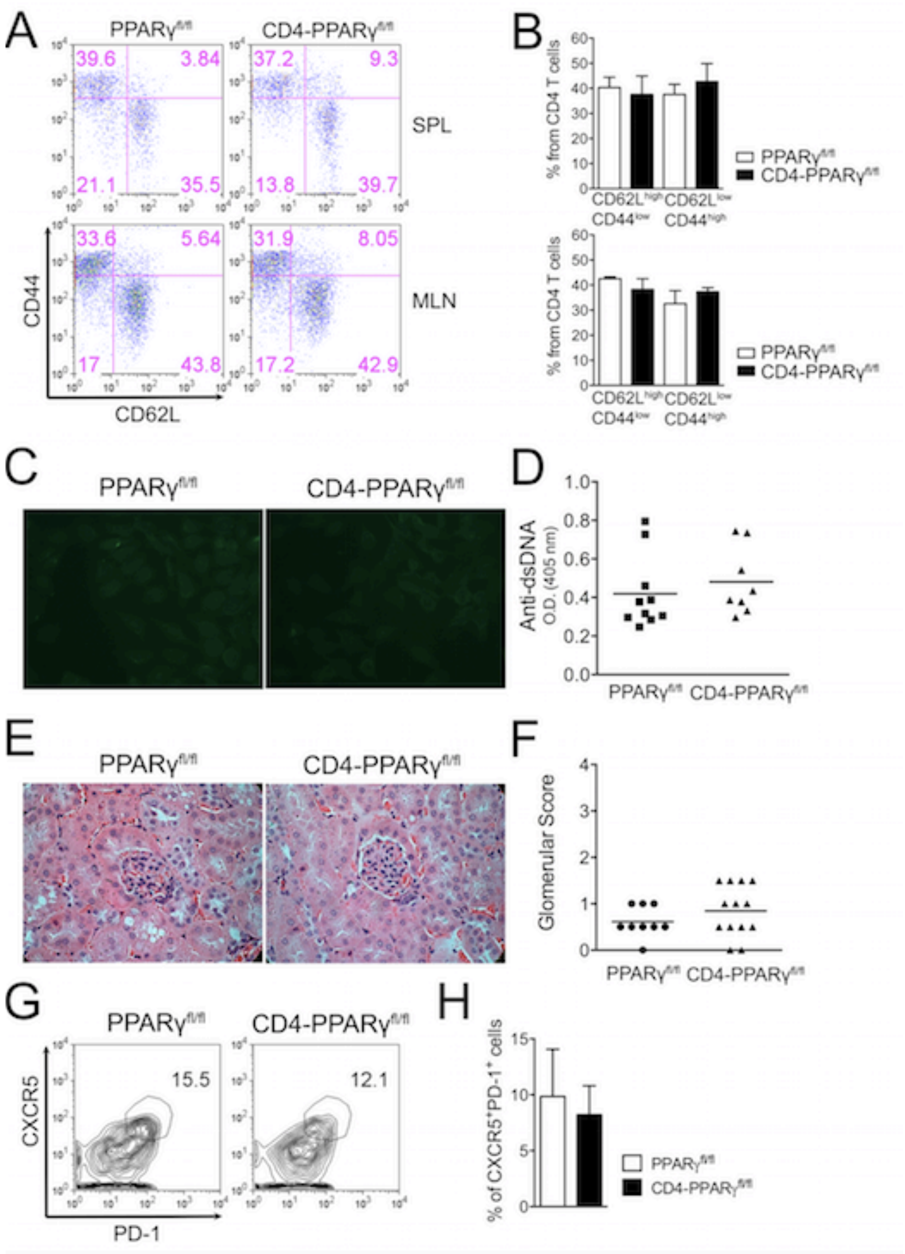

Supplementary Figure S1. **Male CD4-PPARγ^{KO} mice do not develop spontaneous autoimmune phenotypes.** (**a**) Spleens and mesenteric lymph nodes were isolated from 1-year-old littermate control and CD4-PPARγ^{KO} mice. The proportion of naïve (CD62L^{high}CD44^{low}) and activated (CD4+CD62L^{low}CD44^{high}) T cells gated on CD4-positive cells were examined by flow cytometry analysis. (**b**) The % of CD62L^{high}CD44^{low} and CD62L^{low}CD44^{high} T cells gated on CD4-positive cells were

indicated as a bar graph (n=4). (**c**) Spontaneous anti-nuclear antibody (ANA) production was analyzed in the serum of 6-month-old male littermate control and CD4-PPARγ^{KO} mice. Pre-fixed NIH3T3 cells were stained with Alexa Fluor 488-conjugated anti-mouse Ig antibody following staining with diluted sera. (**d**) Anti-dsDNA antibody production was determined in the serum of 1-year-old male littermate control and CD4-PPARγ^{KO} mice by ELISA. Each dot represents a single mouse (n=8-10). (**e**) Glomerular inflammation was analyzed by hematoxylin and eosin (H&E) staining of kidneys from 1-year-old male littermate control and CD4-PPARγ^{KO} mice. (**f**) Glomerular inflammatory score was examined in 1-year-old male littermate control and CD4-PPARγ^{KO} mice (n=9-13). (**g**) Spontaneous induction of T_{FH} cells was analyzed in the spleen from 1-year-old male littermate control and CD4-PPARγ^{KO} mice. (**h**) The % of PD-1^{+}CXCR5^{+} T_{FH} cells was represented as a bar graph. Values shown are means ± SEM (n=4).

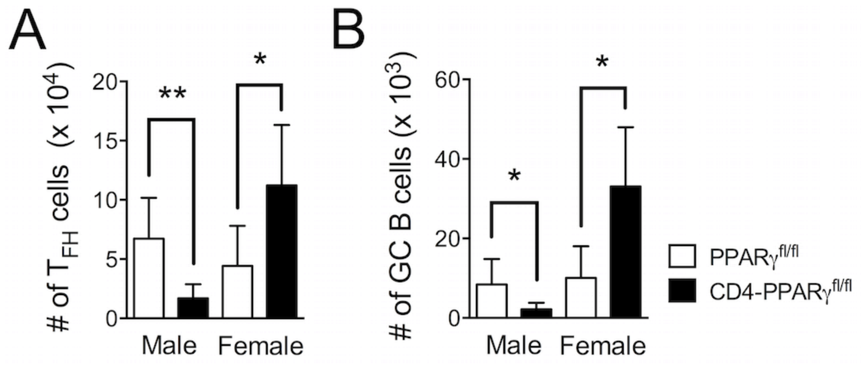

Supplementary Figure S2. Male CD4-PPARγ^{KO} but not female mice have lower numbers of T_{FH} and GC B cells. T_{FH} and GC B cells were induced by immunization of six- to eight-week-old male and female littermate control and CD4-PPARγ^{KO} mice with NP-OVA. Seven days after immunization, the numbers of T_{FH} and GC B cells in lymph nodes were examined. (**a**) Bcl-6 and CXCR5 double-positive cells gated on $CD4^{+}CD44^{high}$ were identified as T_{FH} cells and the number of Bcl-6^{+}CXCR5^{+} T_{FH} cells were analyzed. (**b**) The number of GL-7^{+}CD95^{+} GC B cells gated on B220-positive cells from lymph nodes was analyzed as GC B cells. The data represent means ± SEM (n=4/group, three independent experiments). $*P < 0.05$, $**P < 0.01$ by a two-tailed, unpaired Student's *t*-test.

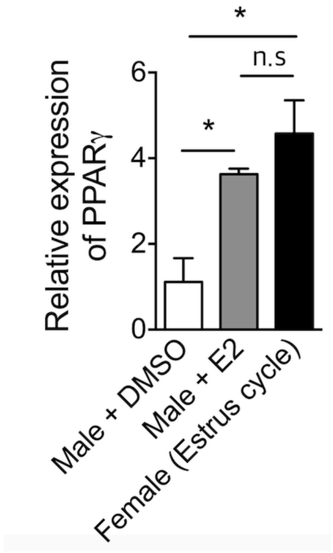

Supplementary Figure S3. **In vivo administration of E2 results in increased PPARγ mRNA expression in male mice.** Six- to eight-week-old male C57BL/6 mice were administrated with DMSO and 60 μg of E2, once a day from day 1 to day 6 and spleens were isolated from the mice at day 7. Menstrual cycle was monitored in six- to eight-week-old female C57BL/6 mice and splenocytes were isolated from the mice at estrus stage. Total RNA was isolated from the splenocytes and the expression level of PPARγ was determined using real-time PCR and was normalized to β-actin. The data shown represent means ± SEM (n=2~3, two independent experiments). *$P < 0.05$ by a two-tailed, unpaired Student's *t*-test.

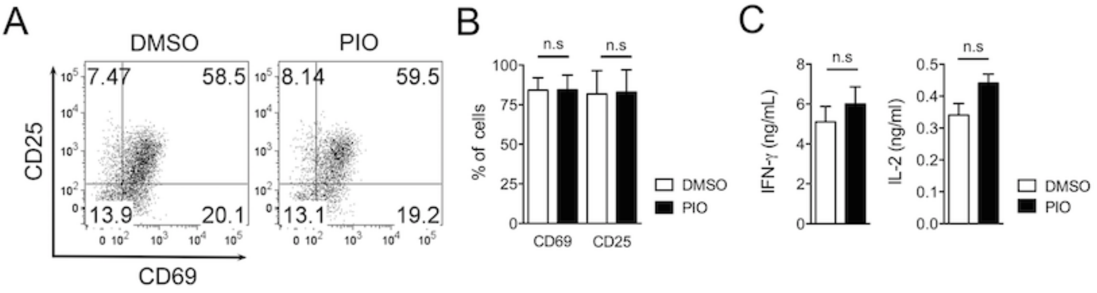

Supplementary Figure S4. **Pioglitazone does not affect T cell activation in male cells.** Splenocytes were isolated from six- to eight-week-old male mice and stimulated with anti-CD3 and anti-CD28 for 24 h in the presence of DMSO or pioglitazone. (**a, b**) CD69 and CD25 expression gated on CD4-positive cells were analyzed with flow cytometry and the % of CD69- and CD25-positive cells was represented as a bar graph (n=6). (**c**) IFN-γ and IL-2 cytokine production levels following TcR stimulation were analyzed by ELISA using cultured supernatant (n=6).

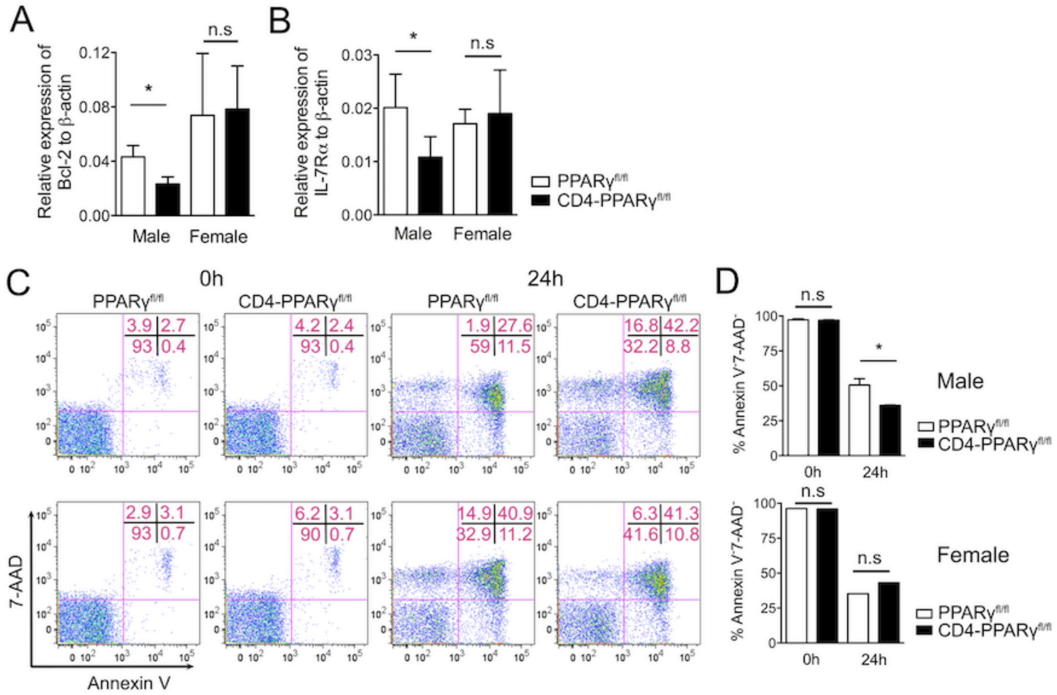

Supplementary Figure S5. **Male PPARγ-deficient T cells are apoptotic following TcR stimulation.** Naïve T cells ($CD4^{+}CD62L^{high}$) were purified from the spleens of male littermate control and CD4-$PPAR\gamma^{KO}$ mice and were stimulated with anti-CD3 and anti-CD28 antibodies for 3 days. (**a**,**b**) Expression levels of Bcl-2 and IL-7Rα in anti-CD3- and anti-CD28-stimulated male and female T cells from littermate control and CD4-$PPAR\gamma^{KO}$ mice were analyzed with real-time PCR and normalized to β-actin (n=4). (**c**,**d**) Naïve T cells were purified from the spleens of male and female littermate control and CD4-$PPAR\gamma^{KO}$ mice. The cells were incubated in serum depleted RPMI media overnight and proportion of live cells (Annexin V^{-}7-AAD^{-}) was examined by flow cytometry analysis. The % of Annexin V^{-}7-AAD^{-} live cells gated on CD4-positive cells. Values shown are means ± SEM (n=3), $*P < 0.05$.
